# Prior COVID-19 vaccination and cognitive outcomes after SARS-CoV-2 infection: a mini-review

**DOI:** 10.1590/1980-5764-DN-2026-0494

**Published:** 2026-10-02

**Authors:** Lucas Gomes da Silva, Jordano César Milagres Oliveira, Bruna Carmeni Di Fini, Aline Arantes Porto, Danielle de Souza Costa, Débora Marques de Miranda, Marco Aurélio Romano-Silva, Jonas Jardim de Paula

**Affiliations:** 1 Universidade Federal de Minas Gerais, Faculdade de Medicina, Programa de Pós-Graduação em Medicina Molecular, Belo Horizonte MG, Brazil.; 2 Universidade Federal de Minas Gerais, Faculdade de Medicina, Instituto Nacional de Ciência e Tecnologia em Neurotecnologia Responsável, Belo Horizonte MG, Brazil.; 3 Universidade Federal de Minas Gerais, Faculdade de Medicina, Departamento de Pediatria, Belo Horizonte MG, Brazil.; 4 Universidade Federal de Minas Gerais, Faculdade de Medicina, Departamento de Psiquiatria, Belo Horizonte MG, Brazil.

**Keywords:** Cognition, Memory Disorders, COVID-19, Post-Acute COVID-19 Syndrome, Vaccination., Cognição, Transtornos da Memória, COVID-19, Síndrome de Pós-COVID-19 Aguda, Vacinação.

## Abstract

COVID-19 infection has been frequently associated with cognitive deficits that persist for months after the acute phase, but the impact of vaccination on preventing these deficits remains uncertain. Evidence suggests that immunization reduces the incidence and severity of post-COVID symptoms, yet few studies have evaluated its influence on cognition using objective measures. To address this gap, a mini-review was conducted using a systematic search strategy, following PRISMA recommendations where applicable, with a protocol registered in PROSPERO (CRD42025636772). Searches were carried out in the PubMed/MEDLINE and Scopus databases, and eligible studies were those that assessed cognition using objective measures and compared vaccinated and unvaccinated individuals at the time of infection. A total of 1,796 records were identified, of which only five met the inclusion criteria. The included studies varied considerably in cognitive assessment tools, study designs, and sample sizes. Four of the five studies found no association between prior vaccination and cognitive deficits, whereas the only study that identified a subtle difference among individuals who had received at least two doses of the vaccine had a substantially larger sample than all the others combined. Methodological inconsistencies in cognitive assessment, variability in the definitions of vaccination status, and the lack of stratification by specific cognitive domains limit the interpretation of the current findings. Although vaccination may play a role in protecting against cognitive deficits following COVID-19 infection, the evidence remains limited and inconclusive. Further studies with standardized definitions of vaccination status and comprehensive objective assessments are needed to clarify this relationship.

## RESUMO

A infecção por COVID-19 tem sido frequentemente associada a déficits cognitivos que persistem por meses após a fase aguda, mas o impacto da vacinação na prevenção desses déficits permanece incerto. Evidências sugerem que a imunização reduz a incidência e a gravidade dos sintomas pós-COVID-19; entretanto, poucos estudos avaliaram sua influência sobre a cognição utilizando medidas objetivas. Para abordar essa lacuna, foi realizada uma mini-revisão utilizando uma estratégia de busca sistemática, seguindo as recomendações PRISMA, quando aplicáveis, com um protocolo registrado no PROSPERO (CRD42025636772). Foram realizadas buscas nas bases de dados PubMed/MEDLINE e Scopus, e foram considerados elegíveis os estudos que avaliaram a cognição por meio de medidas objetivas e compararam indivíduos vacinados e não vacinados no momento da infecção. Foram identificados 1.796 registros, dos quais apenas cinco atenderam aos critérios de inclusão. Os estudos incluídos apresentaram considerável heterogeneidade quanto aos instrumentos de avaliação cognitiva, aos desenhos dos estudos e aos tamanhos amostrais. Quatro dos cinco estudos não encontraram associação entre a vacinação prévia e os déficits cognitivos, enquanto o único estudo que identificou uma diferença sutil entre indivíduos que haviam recebido pelo menos duas doses da vacina apresentou uma amostra substancialmente maior do que todos os demais estudos combinados. Inconsistências metodológicas na avaliação cognitiva, variabilidade nas definições de status vacinal e ausência de estratificação por domínios cognitivos específicos limitam a interpretação dos achados atuais. Embora a vacinação possa desempenhar um papel na proteção contra déficits cognitivos após a infecção por COVID-19, as evidências permanecem limitadas e inconclusivas. São necessários estudos adicionais com definições padronizadas do status vacinal e avaliações cognitivas objetivas abrangentes para esclarecer essa relação.

## INTRODUCTION

COVID-19 is an acute respiratory infection caused by the SARS-CoV-2 virus, whose effects on the body range from asymptomatic presentations to cough, dyspnea, septic shock, and death^1^. Although the COVID-19 pandemic was declared over by the World Health Organization, lasting from 2020 to 2023^2^, many affected individuals continue to experience symptoms months after infection — known as the post-COVID condition^3^.

Although SARS-CoV-2 is transmitted through respiratory pathways, infection has frequently been associated with cognitive deficits that persist for months after the acute phase^4,5^, affecting various functions and directly impacting the quality of life of those affected^6^. The mechanism linking COVID-19 infection to cognitive deficits is not yet fully understood, but neuroinflammation^7^, endothelial dysfunction^8^, cerebral hypoxia^9^, and even viral reservoirs^10^ have been implicated in previous studies.

COVID-19 vaccination represented an important step in mitigating the spread of infections and reducing severe cases and hospitalizations due to COVID-19^11^. In addition, recent meta-analyses highlight the important role of COVID-19 immunization in reducing the incidence of persistent symptoms following the acute phase of infection^12,13^. However, the evidence regarding the role of vaccination in preventing cognitive deficits associated with infection remains uncertain. Most studies evaluating post-COVID conditions assess cognition only through participants’ self-reports — a potentially imprecise approach^14^, as it may be influenced, for example, by the sense of security provided by vaccination. Until recently, no studies combining objective cognitive measures and vaccination had been identified^15^, making it difficult to understand the influence of vaccination on cognition.

This mini-review aimed to investigate the role of vaccination in reducing cognitive deficits associated with COVID-19 infection. To this end, studies were selected in which cognition was assessed through objective methods and evaluated in relation to vaccination status — vaccinated or unvaccinated at the time of infection.

## METHODS

### Search strategy

The protocol for this review was previously registered in the International Prospective Register of Systematic Reviews (PROSPERO) (ID CRD42025636772). In addition, data collection followed the guidelines established by the Preferred Reporting Items for Systematic Reviews and Meta-Analyses (PRISMA)^16^, where applicable.

The systematic search for articles was conducted in the PubMed/MEDLINE and Scopus databases on January 25, 2025, with no language restrictions. No filters were applied during the selection process, and studies of any publication date, language, or study design were considered eligible.

The search aimed to identify studies investigating the relationship between COVID-19 vaccination status at the time of infection and objectively measured cognitive outcomes. The search strategy used was: (“cognit*” OR “neuropsych*” OR “neurocognit*”) AND (“vaccin*” OR “immunizat*”) AND (“COVID-19” OR “SARS-COV-2” OR “long COVID” OR “post-COVID condition”).

### Study selection and eligibility criteria

Two independent reviewers (LGS and JCMO) assessed the titles and abstracts of the identified studies. Inter-rater agreement was calculated using Cohen’s Kappa coefficient, with K=0.648 (standard error – SE=0.082, p<0.001), indicating good agreement between the reviewers^17^. Discrepancies were discussed between the two authors. After title and abstract screening, the pre-selected articles underwent full-text eligibility assessment to determine whether they met the predefined inclusion and exclusion criteria.

All review articles, conference papers, conference abstracts, research protocols, and commentaries were excluded. Studies entirely outside the scope of the review, such as investigations on vaccine intention or hesitancy, were also excluded. Articles based solely on in vitro research or animal models were excluded as well. Additionally, studies that did not address vaccination or cognition were excluded. Articles that assessed cognition exclusively through participant self-report were also excluded from this mini-review.

Since the aim of this review was to understand the role of vaccination in preventing cognitive deficits that occur after COVID-19 infection, studies that classified individuals as vaccinated despite having been infected prior to receiving any vaccine dose were excluded. Eligible studies were required to include one group of unvaccinated individuals (*i.e*., no vaccine dose at the time of first infection) and another group of individuals who had been vaccinated at the time of first infection.

Studies were included if they addressed COVID-19 vaccination and cognition, as measured through objective cognitive tests. Articles were also required to specify the timing of vaccination (before or after infection) to allow for an understanding of the role of vaccination in preventing cognitive deficits.

### Data extraction and synthesis

For the studies included in the qualitative synthesis, information was extracted on study design, data collection period, sample characteristics, cognitive assessment instruments, source and definition of vaccination status, number of doses, infection severity, viral variants, and the main findings regarding the association between vaccination and cognition. Given the marked methodological heterogeneity across studies, the results were synthesized narratively.

## RESULTS

The search strategy identified 2,418 records across the databases. After removing duplicates (n=622), the remaining 1,796 articles underwent title and abstract screening to assess compliance with the inclusion criteria, resulting in the exclusion of 1,727 articles. The remaining studies (n=69) were read in full, and five met all inclusion criteria. The study selection flowchart is presented in Figure 1.

**Figure 1:**
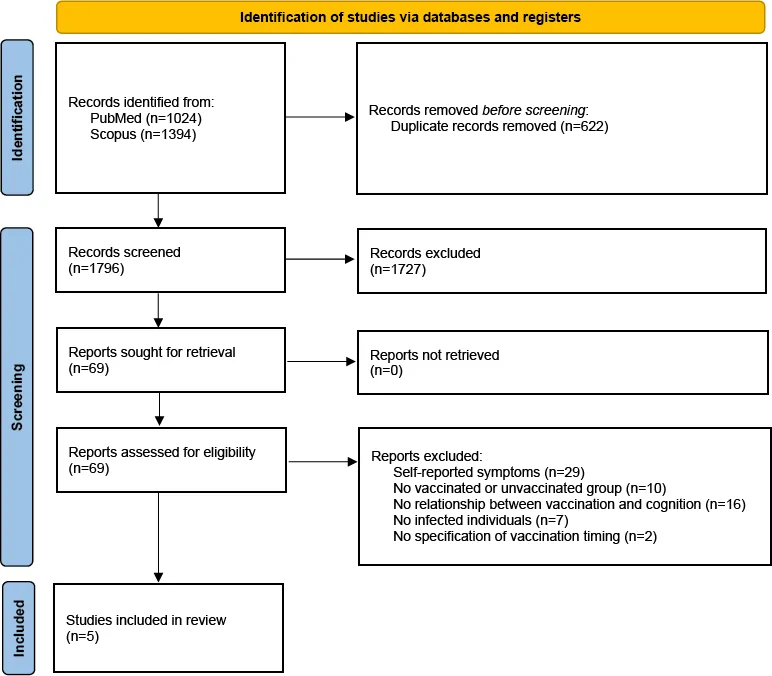
Study Selection Flowchart (Adapted from PRISMA).

Most of the studies excluded during screening were outside the scope of the research, in addition to many reviews, commentaries, and studies on vaccine intention or hesitancy. A significant portion of studies were excluded at the screening stage because they specified that cognitive data were obtained through subjective self-reports, a previously established exclusion criterion.

During the eligibility assessment, most of the excluded articles did not use objective measures of cognitive performance. These were followed by studies that reported vaccination status only as a demographic characteristic of the sample, without examining any correlation between vaccination profile and cognitive outcomes. Only five articles met the inclusion criteria for this mini-review, and a more detailed description of each can be found in Table 1.

**Table 1:** Studies included in the mini-review.

Article Title and Authors	Study Design	Data Collection Period	Sample (n=)	Sample Groups	Tests Used	Vaccination Data Collection	Vaccines	Definition of Vaccinated	Doses	Infection Severity	Variants
Neuropsychiatric post-acute sequelae of COVID-19: prevalence, severity, and impact of vaccination^18^. Alaa Elmazny et al., 2023^18^	Cross-sectional study	May/2020 - February/2022	1,638 participants who developed neuropsychiatric symptoms after infection and met the established criteria for PASC.	Vaccinated and unvaccinated. Partially vaccinated individuals (one dose) were excluded.	MoCA	Not specified.	Pfizer (15.2%); AstraZeneca (31.7%); Johnson & Johnson (3.9%); Sputnik V (1.0%); Sinovac (18.1%); Sinopharm BBIBP (28.5%); Moderna (1.6%).	Unvaccinated: no doses received at the time of infection. Vaccinated: at least two doses administered, with an interval of at least two weeks before the first infection.	At least two doses before infection.	Mild (83.4%), moderate (11.8%), and severe (4.8%).	Not specified.
Factors Associated with Cognitive Impairment in Patients with Persisting Sequelae of COVID-19^19^. Bonner-Jackson et al., 2024^19^	Retrospective observational study	Not specified.	210 participants with PASC and 369 participants in the control group.	The control group included adult patients with cognitive complaints but no objective impairment on tests. The clinical group included patients with PASC (brain fog or cognitive dysfunction persisting for 28 days or more after infection).	WMS-IV Logical Memory, RAVLT, BVMT-R, WRAT-IV Reading, BNT-2, Verbal Fluency, JOL, Digit Span, Matrix Reasoning and Similarities from WAIS-IV, DKEFS, SCWT, TMT, WCST, CCPT-3.	Accessed via electronic medical records.	Not specified.	Unvaccinated: no doses received at the time of infection. Vaccinated: vaccinated at the time of infection. The study does not specify the 14-day period required for seroconversion, for example. It also does not specify whether one, two, or three doses were considered, etc.	Not specified.	Mentions hospitalization during the acute phase.	Not specified.
Cognition and Memory after Covid-19 in a Large Community Sample^20^. Hampshire et al., 2024^20^	Population-based observational study	May/2020 - March/2022	112,964 participants.	Group 1=no COVID-19. Group 2=asymptomatic. Group 3=“short” COVID-19, with symptoms resolved before four weeks. Group 4=“short” COVID-19, with symptoms persisting up to four weeks. Group 5=COVID-19 symptoms lasting up to 12 weeks. Group 6=COVID-19 symptoms lasting more than 12 weeks.	Cognitron Battery (eight cognitive tasks).	Not specified.	Compared Pfizer and AstraZeneca vaccines but did not report whether other vaccines were included or the percentage of participants receiving each vaccine.	Participants were considered vaccinated against SARS-CoV-2 if they had received a vaccine at least 14 days before infection.	Specified different doses (0, 1, or more than one).	Included non-hospitalized individuals, participants who visited the emergency department, those who were hospitalized, and those admitted to the ICU.	Original, Alpha (B.1.1.7), Delta (B.1.617.2), and Omicron (B.1.1.529).
Vaccination prior to SARS-CoV-2 infection does not affect the neurologic manifestations of long COVID^21^. Mukherjee et al., 2025^21^	Prospective observational study	May/2020 - January/2023	200 hospitalized participants and 1,100 non-hospitalized participants.	PNP=hospitalized. NNP=non-hospitalized. PVI=unvaccinated. BTI=vaccinated. In addition, patients were required to have at least six weeks of persistent neurological symptoms following confirmed COVID-19 infection.	NIH Toolbox v2.1	Not specified.	Not specified.	PVI is defined as SARS-CoV-2 infection occurring before any SARS-CoV-2 vaccination. BTI is defined as SARS-CoV-2 infection occurring more than two weeks after receiving any SARS-CoV-2 vaccine.	Apparently, individuals who received only one dose were also considered vaccinated.	Hospitalized and non-hospitalized.	Not specified.
Mental health and chest CT scores mediate the relationship between COVID-19 vaccination status and seroconversion time: A cross-sectional observational study in B.1.617.2 (Delta) infection patients^22^. Zhang et al., 2022^22^	Cross-sectional observational study	July/2021 - September/2021	91 participants infected with the Delta variant.	Unvaccinated (34 participants), partially vaccinated (23 participants), and vaccinated (34 participants).	MMSE	Accessed via electronic medical records.	Only inactivated vaccines (CoronaVac, BBIBP-CorV, and KCONVAC).	Unvaccinated: no doses or a single dose received less than 14 days before infection. Partially vaccinated: received only one dose more than 14 days before infection, or received two doses with an interval of less than 14 days between the second dose and infection. Vaccinated: received two doses of the vaccine, with an interval of more than 14 days between the last dose and infection.	0, 1, and 2 doses.	Mild and moderate.	Only Delta (B.1.617.2), identified by genomic sequencing.

Abbreviations: PASC, post-acute sequelae of SARS-CoV-2 infection; MoCA, Montreal Cognitive Assessment; WMS-IV, Wechsler Memory Scale – Fourth Edition; RAVLT, Rey Auditory Verbal Learning Test; BVMT-R, Brief Visuospatial Memory Test – Revised; WRAT-IV, Wide Range Achievement Test – Fourth Edition; BNT-2, Boston Naming Test – Second Edition; JOL, Judgment of Line Orientation; WAIS-IV, Wechsler Adult Intelligence Scale – Fourth Edition; DKEFS, Delis–Kaplan Executive Function System; SCWT, Stroop Color and Word Test; TMT, Trail Making Test; WCST, Wisconsin Card Sorting Test; CCPT-3, Conners Continuous Performance Test – Third Edition; ICU, intensive care unit; NIH Toolbox v2.1, National Institutes of Health Toolbox, version 2.1; PVI, prevaccination infection; BTI, breakthrough infection; MMSE, Mini-Mental State Examination.

### Study characteristics

The five included studies were conducted in Egypt^18^, the United States^19^, England^20^, the United States^21^, and China^22^. Among those that specified the data collection period, most began collecting data at the onset of the pandemic, around May 2020. Statistical analyses varied across studies, with regression analyses being the most prominent.

Regarding study design, the included articles comprised two cross-sectional studies^18,22^, one retrospective observational study^19^, one prospective observational study^21^, and one population-based observational study nested within the REACT cohort^20^.

### Participant characteristics

Sample sizes varied across the studies. All included articles involved adults aged 18 years or older who had been infected with COVID-19. Most studies further subdivided the sample beyond vaccination status, considering factors such as hospitalization and symptom duration. The included studies required a confirmed COVID-19 diagnosis through reverse transcription polymerase chain reaction (RT-PCR), except for the study by Mukherjee et al.^21^, which also included participants diagnosed using rapid antigen tests, and the study by Hampshire et al.^20^, which accepted participants with self-reported infection.

### Assessment instruments and cognitive domains evaluated

Each of the included studies used a different assessment instrument. The studies by Zhang et al.^22^ and Alaa Elmazny et al.^18^ used only a single screening tool: the Mini-Mental State Examination (MMSE) and the Montreal Cognitive Assessment (MoCA), respectively. The studies by Hampshire et al.^20^ and Mukherjee et al.^21^ conducted online assessments using the Cognitron Battery and the NIH Toolbox, respectively, which allow for a more comprehensive evaluation of multiple cognitive functions. Only the study by Bonner-Jackson et al.^19^ employed an extensive in-person assessment, covering multiple domains in a more controlled research setting. Overall, the cognitive constructs evaluated across the studies included memory, attention, executive functions, language, processing speed, and visuospatial processing.

### Vaccination

Only the studies by Bonner-Jackson et al.^19^ and Zhang et al.^22^ specified how vaccination data were obtained, which was through access to participants’ electronic vaccination records. Regarding vaccine manufacturer and technology, only Alaa Elmazny et al.^18^ and Zhang et al.^22^ reported this information for the entire sample. These were also the only two studies that defined a participant as “vaccinated” if they had received at least two doses of the vaccine and if the interval between the last dose and infection exceeded 14 days. Hampshire et al.^20^ and Mukherjee et al.^21^ considered individuals vaccinated if they had received a vaccine at least 14 days before infection, with the former stratifying participants based on whether they had received only one dose or more than one, and the latter apparently including individuals with just a single dose. Lastly, the study by Bonner-Jackson et al.^19^ did not specify the number of doses received or the interval between the last dose and infection.

### Infection severity and variants

None of the studies included only participants with a specific level of disease severity. Instead, individuals with varying degrees of severity were included, ranging from asymptomatic cases to those hospitalized in intensive care units. Regarding variants, Zhang et al.^22^ conducted genomic sequencing and included only participants infected with the Delta variant (B.1.617.2). Hampshire et al.^20^ inferred the likely variant based on the date of infection and genomic surveillance data.

### Comparisons between vaccinated and unvaccinated individuals

Alaa Elmazny et al.^18^ used the MoCA as an objective criterion to define post-COVID-19 cognitive dysfunction, adopting a cut-off score of <26. In total, 167 participants (10.2%) met this criterion, and all had scores ≥19, consistent with mild cognitive impairment. However, the comparison reported between vaccinated and unvaccinated groups did not refer to objective MoCA performance, but rather to the severity of the symptom “cognitive dysfunction” on a visual analog scale. In this comparison, no statistically significant difference was found between groups (median 4 [IQR: 1–4] vs. 3 [IQR: 3–4], p=0.698).

Bonner-Jackson et al.^19^ conducted a comprehensive in-person neuropsychological assessment covering multiple cognitive domains. The clinical sample was stratified into two groups: cognitive impairment (n=93) and preserved cognition (n=117). Individuals with a T-score below 35 in at least one of the assessed domains were classified as cognitively impaired. The authors compared these subgroups based on vaccination status at the time of infection and found no statistically significant differences in the descriptive analysis (p=0.74) or in the multivariate logistic regression (p=0.72).

The study by Hampshire et al.^20^ used propensity score analysis to reduce bias in the comparison between vaccinated and unvaccinated groups. This technique involves matching individuals with similar characteristics (such as age, comorbidities, and infection period) based on the probability of belonging to one of the groups, calculated through a score. After matching, the authors compared global cognitive performance between the groups. The results showed that participants vaccinated with only one dose did not differ from the unvaccinated group (standardized difference=0.08 standard deviations), whereas those who received more than one dose exhibited a small cognitive advantage compared with the unvaccinated group (standardized difference=0.15 standard deviations). Additionally, the authors compared individuals who received two doses of the Pfizer vaccine with those who received the same number of doses of AstraZeneca and found a non-significant difference in global cognitive score (standardized difference=–0.07 standard deviations).

The study by Mukherjee et al.^21^, in addition to subdividing the sample according to vaccination status at the time of infection, also separated participants based on whether they had been hospitalized due to COVID-19. The results are reported using the median and interquartile range and, unlike the other studies, are presented individually for each cognitive construct assessed by the instrument. In the hospitalized group, no statistically significant differences were found between vaccinated and unvaccinated participants in processing speed (p=0.34), attention (p=0.56), executive functions (p=0.71), and working memory (p=0.82), which are the four domains assessed by the NIH Toolbox. Similarly, no significant differences were found in the non-hospitalized group across any of the four domains: processing speed (p=0.09), attention (p=0.89), executive functions (p=0.98), and working memory (p=0.39).

Lastly, the study by Zhang et al.^22^ found a difference in MMSE performance between vaccinated and unvaccinated groups. Among vaccinated individuals, 5.6% scored below 25 (indicative of cognitive impairment), whereas this same finding was observed in 26.5% of the unvaccinated group. However, the authors noted that this difference was reduced after adjusting for age (p=0.013) and lost statistical significance when adjusted for sex, age, and education level (p=0.076).

## DISCUSSION

Despite the large number of publications on COVID-19 and cognition, this mini-review shows that very few studies have addressed the influence of vaccination on cognitive deficits measured using objective instruments; only five articles were identified. Although no language restrictions were applied, all included articles were published in English. Each included study was published in a different journal, but all were peer-reviewed.

Most of the studies included in this mini-review indicate that prior COVID-19 vaccination did not influence the cognitive outcomes of infected participants. Only the study by Hampshire et al.^20^, which involved a large sample, found a subtle difference between vaccinated and unvaccinated individuals. However, this result should be interpreted in light of the significant limitations present in the included studies.

In the study by Alaa Elmazny et al.^18^, the use of an objective cognitive measure represents an important strength, especially in a field in which many studies rely exclusively on subjective complaints. However, cognition was assessed only with the MoCA, and post-COVID-19 cognitive dysfunction was defined using a fixed cut-off score of <26^18^. This approach deserves caution, since performance on brief cognitive screening instruments may be influenced by educational level and may not adequately capture subtle deficits in heterogeneous post-COVID populations^23,24^. Therefore, the use of a single cut-off may have affected the estimated frequency of cognitive dysfunction and limited the interpretation of comparisons between vaccinated and unvaccinated individuals. Moreover, although the authors emphasized differences in the severity of some neuropsychiatric symptoms according to vaccination status, the MoCA findings themselves were not substantially explored in the discussion. This limits the interpretation of the cognitive results and reinforces the need for more comprehensive and educationally sensitive cognitive assessments in future studies.

In the study by Bonner-Jackson et al.^19^, vaccination data were not discussed by the authors, and no hypotheses were proposed to explain the non-significant finding. Although the dichotomization of the sample into cognitively impaired and preserved groups may help simplify the data, the chosen strategy may have compromised important aspects related to the specificity of cognitive deficits. Grouping all participants with impairments into a single category prevents the analysis of vaccination effects on specific domains, such as visuospatial processing and memory, making it difficult to assess the impact of vaccination on different cognitive functions.

Moreover, the sampling in the study by Bonner-Jackson et al.^19^ presents inconsistencies that hinder the interpretation of the results. Initially, it is suggested that the control group consisted of individuals with cognitive complaints who had not been infected with COVID-19, as stated in the study’s objectives. However, throughout the discussion, it becomes apparent that the control group had in fact been infected, with over 30% of participants in this group presenting moderate to severe symptoms, although they apparently did not meet the study’s criteria for post-acute sequelae of SARS-CoV-2 infection (PASC; *i.e*., persistence of cognitive symptoms for at least 28 days). It is unclear how, in fewer than 28 days, participants could have noticed cognitive symptoms and promptly undergone a comprehensive neuropsychological battery, particularly following a severe respiratory infection. Based on this premise, the preserved cognition subgroup within the clinical sample appears more comparable to the control group than to the cognitively impaired group, making the interpretation of the results confusing and potentially biased. Finally, the definition of “vaccinated” versus “unvaccinated” is vague, lacking detail about the number of doses required to be considered vaccinated and with no mention of the necessary time for seroconversion after administration of the second dose, for instance.

The study by Hampshire et al.^20^ analyzed a large sample of participants and conducted multiple analyses considering variables beyond vaccination, such as number of reinfections, symptom persistence, and viral variants. Despite the lack of control over the testing environment, since the assessments were conducted online using the participants’ own devices, the large sample reinforces the relevance of the findings.

However, as clearly stated in the article’s supplementary table, the authors considered vaccination status based on the most severe (*i.e*., longest-lasting) infection, rather than the first infection. Thus, a participant who experienced an initial infection while unvaccinated and a subsequent infection after vaccination would be classified as “vaccinated.” This definition may introduce bias into the interpretation of results, as the impact of vaccination may be underestimated or confounded by the effects of multiple infections.

Additionally, Hampshire et al.^20^ used factor analysis to derive a global cognitive score based on performance across eight tasks from the Cognitron Battery. As a result, like the study by Bonner-Jackson et al.^19^, the authors did not break down cognition by specific domains, making it impossible to conduct a more detailed analysis of the effects of vaccination on different cognitive constructs.

The study by Mukherjee et al.^21^ offers an individualized analysis of each cognitive domain assessed, correlating it with vaccination status, a notable distinction from the studies by Bonner-Jackson et al.^19^ and Hampshire et al.^20^ However, an important consideration when interpreting the results is the lack of clarity regarding the number of vaccine doses required for classification as “vaccinated.” It is unclear whether, for example, receiving only one dose prior to infection would place a participant in the vaccinated group. If cognitive protection depends on booster doses, as suggested in the study by Hampshire et al.^20^, the classification used by Mukherjee et al.^21^ may overlook the possibility that the protective effect of vaccination is dose-dependent, highlighting the need for more detailed criteria. This concern is especially relevant given the country in which the study was conducted, where there is a 10% drop in the number of individuals completing the primary vaccination series, and only 36% of the population has received at least one booster dose^25^.

Moreover, although the NIH Toolbox is a practical instrument, it does not assess several important cognitive domains, such as memory, language, and visuospatial processing, which have frequently been reported as impaired following infection.

The study by Zhang et al.^22^ is methodologically well-structured, with clearly defined criteria and comprehensive information provided to aid in the interpretation of the results. However, cognition was assessed only with the MMSE, and cognitive impairment was defined using a cut-off of <25^22^. Although a higher proportion of unvaccinated participants met this criterion, the difference in MMSE scores was no longer statistically significant after adjustment for age, sex, and education^22^. This finding reinforces the need for caution when interpreting the cognitive results, especially because the MMSE is a brief screening instrument with limited sensitivity for subtle deficits and restricted coverage of domains that are particularly relevant in post-COVID conditions, such as attention and executive functioning^23,24^. In addition, the primary focus of the study was not cognition itself, but rather the relationships among vaccination status, chest CT findings, mental health, quality of life, and seroconversion time^22^. Therefore, although the study contributes useful data, its design and cognitive assessment strategy limit stronger conclusions regarding the protective role of vaccination against post-COVID cognitive impairment.

The sample size of each study must be considered when interpreting the results. While the study by Zhang et al.^22^ included 91 participants, the study by Hampshire et al.^20^ included 112,964 participants. The fact that only one of the five included studies found a statistically significant difference should be interpreted in light of the sample size of that study, which consisted of a notably large number of participants.

Across the included studies, only Mukherjee et al.^21^ examined vaccination effects according to individual cognitive domains, whereas the remaining studies relied on screening cutoffs or global cognitive scores. This limited domain-specific detail may have obscured selective associations between vaccination status and specific cognitive functions, particularly in domains that have been repeatedly implicated in post-COVID conditions.

Another important point is that Hampshire et al.^20^ found a difference only among individuals who had received more than one dose of the vaccine, which may explain the absence of differences between vaccinated and unvaccinated groups in other studies included in this mini-review, such as those by Mukherjee et al.^21^ and Bonner-Jackson et al.^19^, which did not define “vaccinated” exclusively as individuals who had received at least two doses of the vaccine 14 days prior to infection. This finding is supported by a meta-analysis published in 2023^12^, which reported that receiving two doses of the vaccine prior to infection reduces the incidence of post-COVID conditions compared with unvaccinated individuals and those vaccinated with only one dose.

Viral variants appear to play a distinct role in the symptomatology of the infection^26^. Nevertheless, only the studies by Hampshire et al.^20^ and Zhang et al.^22^ included variants as study variables, representing a relevant limitation of the findings.

Unfortunately, none of the included studies conducted analyses of biomarkers associated with the persistence of cognitive deficits after infection, despite this being widely discussed in the literature^27,28,29^. Since prior vaccination has been linked to a reduction in the pro-inflammatory cytokine storm^30^, it would be valuable to investigate whether a decrease in the inflammatory response correlates with cognitive symptoms, thereby enabling a better understanding of post-infection cognitive deficits. For instance, the study by Vanderheiden et al. (2024)^31^ found that prior vaccination reduced IL-1β levels and, consequently, memory deficits following COVID-19 infection in mice.

Future studies should investigate whether the effect of vaccination varies across different cognitive functions, allowing for a more detailed understanding of the neuroprotective impact of immunization. Another possibility is for already completed studies to conduct stratified analyses by cognitive construct to examine whether vaccinated individuals differ from unvaccinated individuals across specific domains.

These findings should be interpreted with caution, as the five included studies were highly heterogeneous in several key aspects. The selected populations ranged from hospitalized patients infected with the Delta variant^18^ to large community-based samples and patients evaluated in specialized Neuro-COVID clinics^20,21,22^. In addition, the definition of post-COVID condition/PASC was not uniform across studies, varying according to symptom-duration thresholds, clinical setting, and case-ascertainment procedures^18,19,21^. Vaccination status was also inconsistently defined, with differences in the number of doses required, the interval considered necessary for seroconversion, and the handling of partially vaccinated individuals^18,20,21,22^. Furthermore, cognitive assessment strategies varied substantially, ranging from brief screening tools such as the MMSE^22^ and MoCA^18^ to online batteries^20–21^ and comprehensive in-person neuropsychological evaluations^19^. Finally, cognitive outcomes were operationalized and reported differently across studies, including categorical impairment cutoffs, domain-specific analyses, and global cognitive scores^18–22^. Taken together, this heterogeneity limits direct comparability across studies and restricts the generalization of current findings regarding a possible protective effect of vaccination on post-COVID cognitive outcomes.

Given these limitations, further research is needed to clarify the effects of vaccination on post-COVID-19 cognitive deficits. Only five studies met the inclusion criteria for this review, and a substantial number of investigations collected vaccination data only as a demographic characteristic, without specifically examining its association with cognitive outcomes. Since these data have already been collected, it would be both methodologically feasible and highly relevant to perform additional analyses that stratify samples based on vaccination status at the time of infection, as done by Mukherjee et al^21^. This approach would reduce the need for new studies by taking advantage of existing data — particularly from results collected before the start of vaccination campaigns in 2020, when individuals were infected without having received any vaccine doses.

The growing number of studies highlighting the importance of vaccination in reducing cognitive deficits may serve as a valuable tool for health promotion and for encouraging adherence to vaccination campaigns, especially booster doses, which have low uptake in some countries but appear to positively influence outcomes. Moreover, understanding the role of vaccination in the reduction (or not) of cognitive symptoms following infection may contribute to clarifying the mechanisms underlying the cognitive deficits observed in individuals infected with COVID-19, particularly by examining the influence of the immune response and investigating hypotheses such as neuroinflammation.

In conclusion, this mini-review revealed the limited number of studies analyzing the impact of vaccination on cognitive preservation following COVID-19 infection, with only five articles meeting the predefined inclusion criteria. Most of the included studies did not find a significant association between prior vaccination and post-infection cognitive performance. Only one study identified a subtle effect of vaccination in individuals who had received at least two doses. However, the various methodological limitations of the included studies restrict the generalizability of the findings, highlighting the importance of establishing a consensus on the definition of “vaccinated” in studies that use objective cognitive measures.

## Data Availability

The data that support the findings of this study are available from the corresponding author upon reasonable request.
